# Lipopolysaccharides promote pulmonary fibrosis in silicosis through the aggravation of apoptosis and inflammation in alveolar macrophages

**DOI:** 10.1515/biol-2020-0061

**Published:** 2020-08-18

**Authors:** Shiyi Tan, Shang Yang, Mingke Chen, Yurun Wang, Li Zhu, Zhiqian Sun, Shi Chen

**Affiliations:** Key Laboratory of Molecular Epidemiology of Hunan Province, Hunan Normal University, Changsha, 410013, Hunan, China; Department of Pneumoconiosis, Beidaihe Sanitarium for China Coal Miners, Beidaihe, 066100, Hebei, China; Key Laboratory of Molecular Epidemiology of Hunan Province, Hunan Normal University, No. 371 Tongzipo Road, Changsha, 410013, Hunan, China

**Keywords:** silicosis, alveolar macrophages, LPS, apoptosis

## Abstract

Alveolar macrophages (AMs) play an important defensive role by removing dust and bacteria from alveoli. Apoptosis of AMs is associated with lung fibrosis; however, the relationship between this apoptotic event and environmental factors, such as the presence of lipopolysaccharides (LPSs) in the workplace, has not yet been addressed. To investigate whether exposure to LPS can exacerbate fibrosis, we collected AMs from 12 male workers exposed to silica and incubated them in the presence and absence of LPS for 24 h. We show that the levels of cleaved caspase-3 and pro-inflammatory cytokines interleukin (IL)-1β, IL-6, and tumor necrosis factor-alpha were increased in these AMs following LPS treatment. Moreover, we demonstrate that LPS exposure aggravated apoptosis and the release of inflammatory factors in AMs in a mouse model of silicosis, which eventually promoted pulmonary fibrosis. These results suggest that exposure to LPS may accelerate the progression of pulmonary fibrosis in silicosis by increasing apoptosis and inflammation in AMs.

## 1 Introduction

Silicosis, characterized by extensive pulmonary nodular fibrosis, is the most common type of pneumoconiosis [1] and is caused by the prolonged exposure to a high quality of free silica dust [2]. Numerous studies have reported that silicosis can further trigger the development of other pulmonary diseases including tuberculosis and lung cancer [3], posing a serious threat to the health of silica-exposed workers [4]. Accordingly, it is imperative that novel strategies be developed for the prevention of silicosis.

Alveolar macrophages (AMs) play an important defensive role in the progression of silicosis [5]. Normally, when invading lung tissue, silica dust will be phagocytosed by activated AMs; however, since AMs are unable to adequately dissolve the silica dust, apoptosis is triggered, causing the release of a large amount of inflammatory and fibrotic factors. Subsequently, the released SiO_2_ is captured by other AMs, and this process is repeated, finally leading to the aggravation of silicosis [6,7,8].

Lipopolysaccharides (LPSs) are characteristic components of Gram-negative bacterial cell walls, many types of which have been detected in the air of coal mines in China [9]. There is increasing evidence that LPS can aggravate a variety of diseases, such as Alzheimer’s disease and Parkinson’s disease, reproductive system damage, and liver toxicity, by promoting apoptosis and inflammation [10,11,12,13]. Moreover, a previous study detected LPS in the bronchoalveolar lavage fluid of silicosis patients [9]. Accordingly, there is an urgent need to elucidate whether LPS can stimulate the progression of pulmonary fibrosis in silicosis.

Previous studies have shown that apoptosis of AMs is closely correlated with the pathological changes of pulmonary fibrosis [14]; however, the relationship between LPS and apoptosis of AMs in silicosis has not yet been identified.

In the present study, we found that LPS treatment increased apoptosis and the release of inflammatory factors in AMs from silica-exposed workers, in addition to aggravating pulmonary fibrosis in a mouse model of silicosis. Therefore, we speculate that LPS exposure may exacerbate pulmonary fibrosis in silicosis through the aggravation of apoptosis and inflammation of AMs, which may provide a basis for the development of novel preventive strategies for pulmonary fibrosis.

## 2 Materials and methods

### 2.1 Subjects

Twelve male silica-exposed workers were selected for the present study and divided into two groups: six observers, whose X-ray images showed uncertain silicosis-like changes, the nature and severity of which did not dramatically change within 5 years; and six silicosis patients, whose disease was identified by X-ray images. The occupational category of the selected workers was tunneling, during which they were exposed to silica only. Silicosis was diagnosed by a local pneumoconiosis diagnostic group, according to the GBZ70-2015 standard issued in China and the ILO-2000 guidelines. All subjects underwent massive whole-lung lavage at the Beidaihe Sanatorium for Chinese Coal Miners between July and September 2019.


**Informed consent:** Informed consent has been obtained from all individuals included in this study.
**Ethical approval:** The research related to human use has been complied with all the relevant national regulations, institutional policies and in accordance with the tenets of the Helsinki Declaration and has been approved by the Medical Ethics Committee of Hunan Normal University (permit number: hunnu-2016-41).

### 2.2 Reagents

The following reagents were used in this study: Natural crystalline silica particles (Min-U-Sil 5 ground silica; size distribution: 97% <5 µm diameter, 80% <3 µm diameter; median diameter: 1.4 µm) were obtained from the US Silica Company (Frederick, MD, USA). LPS (0111:B4) was purchased from Sigma-Aldrich Company (L3024; St. Louis, MO, USA). Cleaved caspase-3 antibody was purchased from Beyotime Biotechnology Company (AC033; Shanghai, China). Collagen I (Col-1) (ab90395) and alpha smooth muscle actin (α-SMA) (ab5694) antibodies were purchased from Abcam (Cambridge, MA, USA). β-Actin antibody was purchased from Santa Cruz Biotechnology, Inc. (sc-130301; Dallas, TX, USA).

### 2.3 Animals and treatment

Male C57BL/6 mice (18–22 g, 6–8 weeks old) were purchased from the Shanghai Laboratory Animal Center (Shanghai, China). In the present study, 30 mice were randomly divided into 3 treatment groups (*n* = 10) as follows: (1) control group, direct oral-tracheal instillation of 50 µL of sterile saline; (2) crystalline silica group (silica), direct oral-tracheal instillation of 50 µL of aqueous suspension of 3 mg silica crystals in sterile saline; and (3) crystalline silica plus LPS group (silica + LPS), direct oral-tracheal instillation of 50 µL of aqueous suspension of 3 mg silica crystals in 100 µg/mL LPS/sterile saline. The mice were sacrificed at day 28 under anesthesia. Lung tissues were extracted carefully for further study.


**Ethical approval:** The research related to animal use has been complied with all the relevant national regulations and institutional policies for the care and use of animals and has been approved by the Animal Care and Use Committee of Hunan Normal University.

### 2.4 AM isolation, purification, and culture

All subjects underwent a large-capacity lung lavage under general anesthesia. Lavage fluids were collected and filtered through a double-layered gauze to remove mucus, centrifuged at 1,500 rpm, and washed three times with phosphate-buffered saline (PBS) buffer. After cells were counted using a hemocytometer, 5 × 10^6^ cells were seeded in Dulbecco’s modified Eagle’s medium (Gibco/Life Technologies/Thermo Fisher Scientific, CA, USA) supplemented with 10% fetal bovine serum (Invitrogen/Life Technologies/Thermo Fisher Scientific, CA, USA) under 5% CO_2_ at 37°C for 2 h. The nonadherent (non-AM) cells were removed, fresh medium was added, and AMs were incubated at 37°C for a further 24 h as a control group. The cells in the LPS group were incubated in medium containing 1 µg/mL LPS for 24 h. Mouse AMs were collected from bronchoalveolar lavage fluid as described previously [15]. Harvested AMs and supernatants were stored at −80°C until use.

### 2.5 ELISA assay

The levels of interleukin (IL)-1β, IL-6, and tumor necrosis factor-alpha (TNF-α) in the supernatant from AMs were measured by ELISA (R&D Systems, Minneapolis, MN, USA) according to the manufacturer’s instructions. Briefly, blanks, standards, and samples were added separately to a 96-well plate, with two replicates per sample. After mixing by gentle shaking, the plates were incubated for 30 min at 37°C, washed five times with PBS, and 50 mL of HRP-conjugated reagent was added to each well. Following incubation for 30 min at 37°C, the cells were washed and incubated with a mixture of chromogen solutions A and B for 10 min. Stop solution was then added to each well to end the reaction. Blank wells were set to zero, and the optical density of each well at 450 nm was measured within 15 min.

### 2.6 Western blotting

AMs were lysed in cell lysis buffer (Cell Signaling Technology, Danvers, MA, USA) containing 1 mM phenylmethylsulfonylfluoride (Solarbio, Beijing, China), and total protein was quantitated using a BCA Protein Assay kit (Biotechnology, Jiangsu, China). Total protein was separated by sodium dodecyl sulfate–polyacrylamide gel electrophoresis and transferred to polyvinylidene fluoride membrane (Merck Millipore, USA) via a semi-dry electrophoretic method. Membranes were blocked for 1 h in 7% skim milk/PBS and subsequently incubated overnight at 4°C with primary antibodies against cleaved caspase-3, Col-1, and α-SMA. The next day, membranes were washed in PBST and incubated with an HRP-conjugated secondary antibody. Protein expression levels were visualized on X-ray film using an ECL kit and analyzed by the Quantity One 7.0 imaging analysis software. β-Actin was used as an internal control.

### 2.7 Hydroxyproline (HYP) content assay

The HYP content of the middle and lower sections of the prominent lobe of the right mouse lung was determined at day 28 post-surgery using a well-accepted assay [16].

### 2.8 Histology

Inflammation and fibrosis were assessed by hematoxylin and eosin (H&E) or Masson’s trichrome staining of paraffin lung sections (5 µm), according to the manufacturer’s protocol. Fibrosis was scored using the Image-Pro Plus version 6.0 software (Media Cybernetics, Rockville, MD, USA). Three different fields within the middle section of the left lung in three sections per animal (*n* = 4 mice/group) were evaluated to obtain a mean value.

### 2.9 Statistics

All values represent mean ± SD. The SPSS v.19.0 software (SPSS Inc., Chicago, IL, USA) was used for all statistical analyses. The differences between values were evaluated using one-way analysis of variance (ANOVA) followed by pairwise comparisons using a Student–Newman–Keuls *post hoc* test. *P* < 0.05 was considered statistically significant.

## 3 Results

### 3.1 LPS increases apoptosis of AMs from silicosis patients

**Figure 1:**
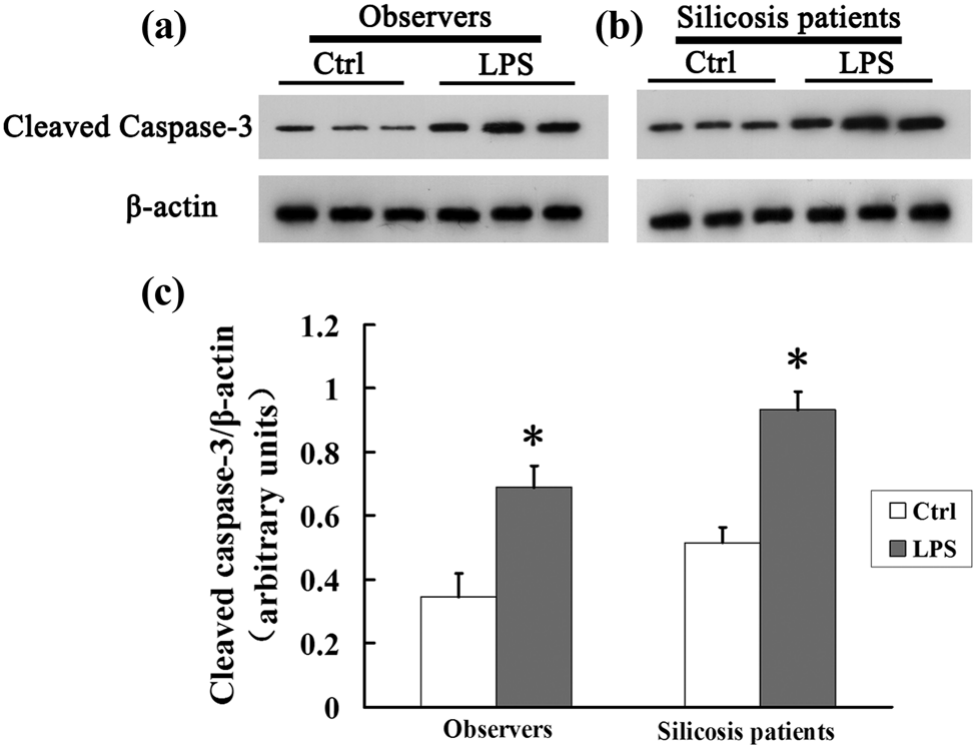
LPS increases the expression of cleaved caspase-3 in AMs from silicosis patients. (a and b) AMs from observers or silicosis patients in the presence or absence of LPS were analyzed for cleaved caspase-3 expression by western blotting; β-actin was used as a loading control. (c) Cleaved caspase-3/β-actin for each group. Significance was determined using one-way ANOVA (*n* = 6. *P < 0.05 vs control).

Caspase-3 is responsible for mediating the terminal signaling pathway of apoptosis [17]. We found that the cleaved caspase-3 level was increased after LPS stimulation in both the observer and silicosis patient groups (Figure 1; *P* < 0.05). These results confirm that LPS aggravated the apoptotic activity in AMs from silicosis patients.

### 3.2 LPS induces the release of IL-1β, IL-6, and TNF-α in AMs from silicosis patients

**Figure 2:**
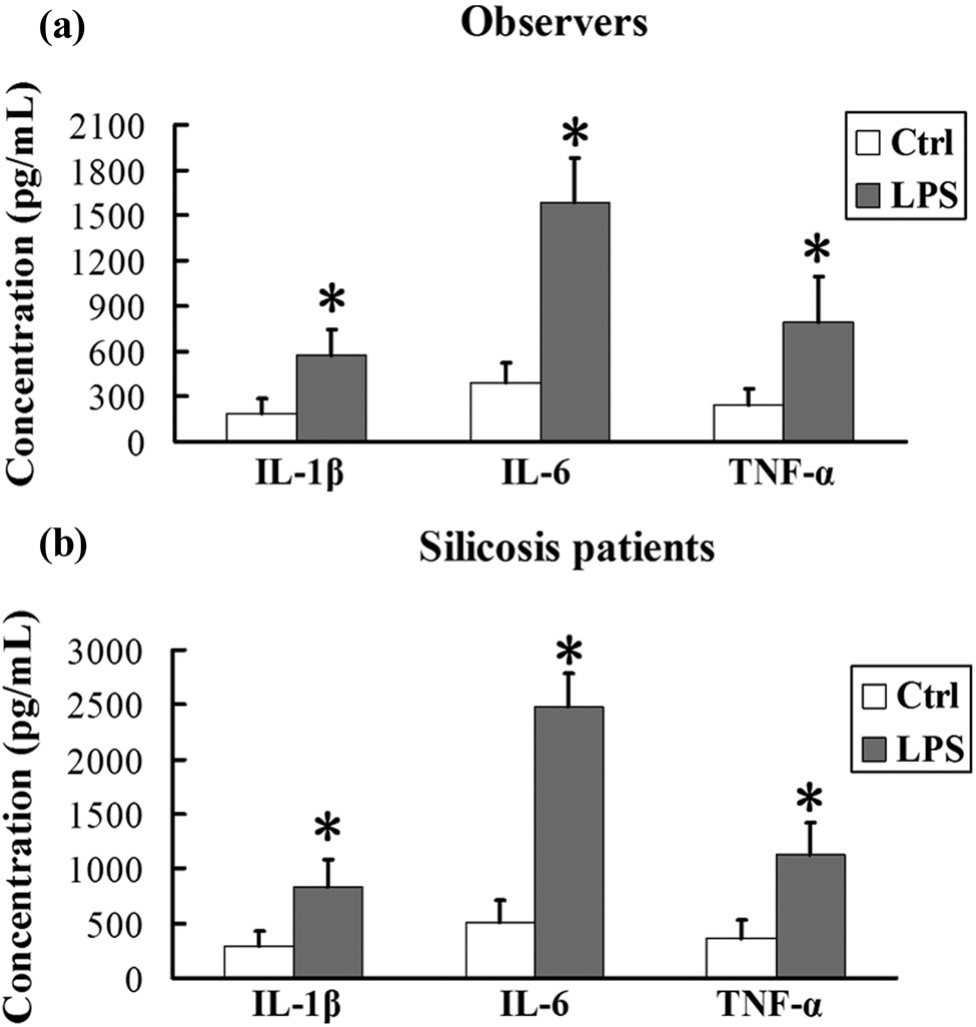
LPS induces the release of IL-1β, IL-6, and TNF-α in AMs from silicosis patients. (a and b) AMs from observers or silicosis patients in the presence or absence of LPS were analyzed for IL-1β, IL-6, and TNF-α levels using an ELISA assay. Significance was determined using one-way ANOVA (*n* = 6. *P < 0.01 vs control).

In AMs from observers and silicosis patients, we found that the concentrations of IL-1β, IL-6, and TNF-α were significantly higher in the presence of LPS than those in the absence of LPS (Figure 2; *P* < 0.01). These results show that LPS aggravated the inflammatory response in silicosis patients.

### 3.3 LPS increases apoptosis of AMs from the mouse silicosis model

**Figure 3:**
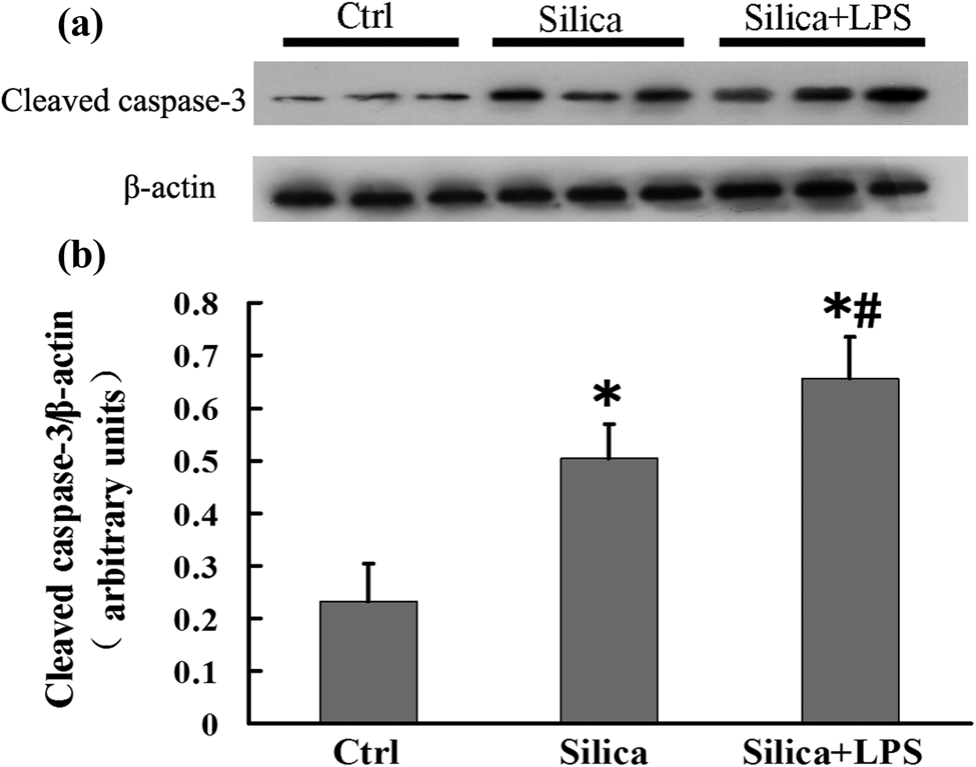
LPS increases the expression of cleaved caspase-3 in AMs from the mouse silicosis model. (a) Mouse AMs from groups treated with sterile saline, silica, and silica + LPS were analyzed for cleaved caspase-3 expression by western blotting; β-actin was used as a loading control. (b) Cleaved caspase-3/β-actin for each group. Significance was determined using one-way ANOVA (*n* = 6 for each group. *P < 0.05 vs control; ^#^ *P* < 0.05 vs silica).

We further tested the relationship between LPS and apoptosis in AMs from the mouse silicosis model. In comparison with the control group, the expression of cleaved caspase-3 was significantly increased in the silica group. Meanwhile, the cleaved caspase-3 level in the silica + LPS group was significantly higher than that in the other two groups, suggesting that LPS aggravated the apoptotic activity in AMs from the mouse silicosis model (Figure 3; *P* < 0.05 for all).

### 3.4 LPS induces the release of IL-1β, IL-6, and TNF-α in AMs from the mouse silicosis model

**Figure 4:**
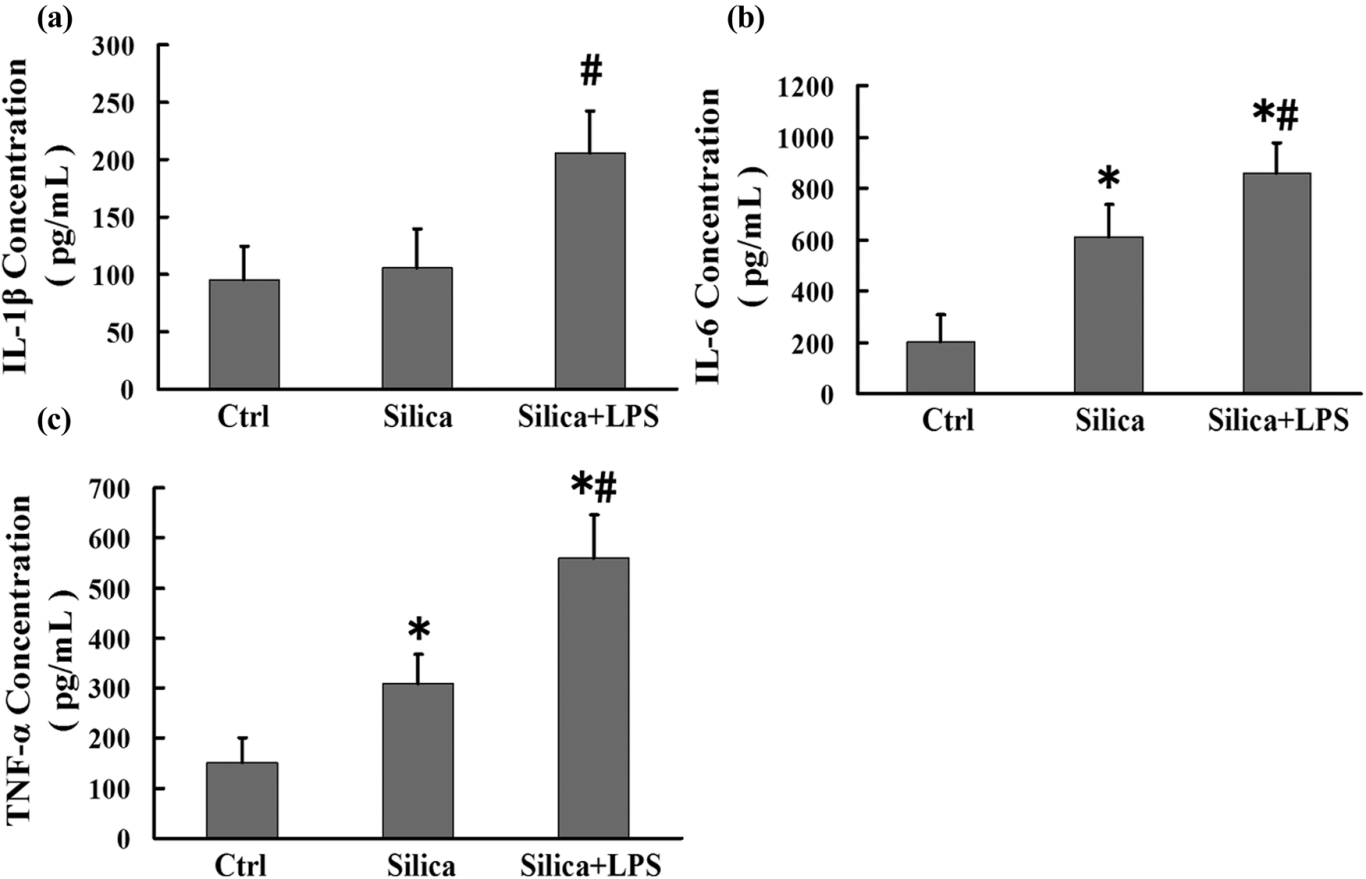
LPS induces the release of IL-1β, IL-6, and TNF-α in AMs from the mouse silicosis model. (a–c) Mouse AMs from groups treated with sterile saline, silica, and silica + LPS were analyzed for IL-1β, IL-6, and TNF-α levels by ELISA. Significance was determined using one-way ANOVA (*n* = 6 for each group. *P < 0.01 vs control; ^#^ *P* < 0.01 vs silica).

We further investigated whether LPS exacerbated the release of pro-inflammatory factors in AMs. We found that the levels of IL-6 and TNF-α in the silica group were significantly higher than those in the control group. Moreover, IL-1β, IL-6, and TNF-α levels in the silica + LPS group were significantly higher than those in the silica group (Figure 4; *P* < 0.01 for all). These results demonstrate that LPS exacerbated inflammation in the mouse silicosis model.

### 3.5 LPS aggravates pulmonary fibrosis in the mouse silicosis model

**Figure 5:**
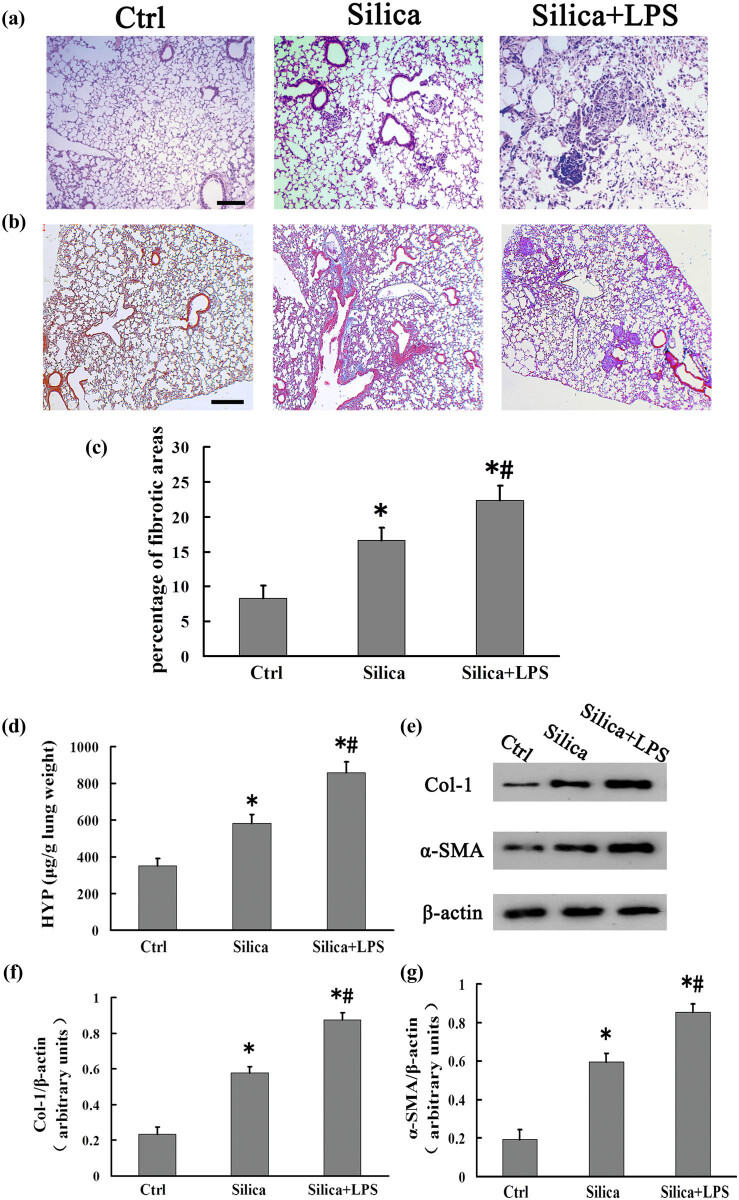
LPS aggravates pulmonary fibrosis in silicosis model mice. (a) H&E staining of mouse lungs on day 28 (scale bar = 100 µm; *n* = 6). (b) Masson’s trichrome staining of mouse lungs on day 28 (scale bar = 200 µm; *n* = 4). (c) Fibrotic score analysis of lung sections on day 28 post-CS instillation. The fibrotic area is presented as a percentage. Data are presented as mean ± SD. Significance was determined using one-way ANOVA (*n* = 4 for each group. *P < 0.05 vs control; ^#^ *P* < 0.05 vs silica). (d) Mouse lung tissues from groups treated with sterile saline, silica, and silica + LPS were analyzed on day 28 for the HYP content. Significance was determined using one-way ANOVA (*n* = 6 for each group. *P < 0.05 vs control; ^#^ *P* < 0.05 vs silica). (e) Mouse lung tissues from groups treated with sterile saline, silica, and silica + LPS were analyzed on day 28 for Col-1 and α-SMA expression by western blotting; β-actin was used as a loading control. (f and g) Col-1/β-actin and α-SMA/β-actin for each group. Significance was determined using one-way ANOVA (*n* = 6 for each group. *P < 0.05 vs control; ^#^ *P* < 0.05 vs silica).

Subsequently, we explored whether LPS can aggravate pulmonary fibrosis in silicosis. After saline injection, there was infiltration of several inflammatory cell types and slight inflammation, but the alveolar structures in the mouse lung tissue were intact. Following silica treatment, further inflammatory cell infiltration and focal interstitial inflammation appeared, which were accompanied by the formation of cellular nodules in the mouse lung tissue. However, in comparison with the silica group, the infiltration and aggregation of inflammatory cells were greater, the focal interstitial inflammation was further aggravated, and a larger number of cellular nodules appeared in the silica + LPS group (Figure 5a). Moreover, we evaluated the Masson’s trichrome-stained left lung middle sections and found that the fibrotic area in the silica + LPS group was significantly larger than that in the silica and control groups (Figure 5b and c). HYP is regarded as a biochemical marker for the degree of collagen deposition [18]. We also measured the levels of Col-1 and α-SMA in the mouse lung tissue. In comparison with the lung tissue from mice treated with saline, the levels of HYP, Col-1, and α-SMA in the lungs of mice treated with silica were significantly increased. Moreover, the levels of these markers in the silica + LPS group were significantly higher than those in the silica group (Figure 5d–g; *P* < 0.05 for all). Taken together, these data suggest that LPS exacerbated pulmonary fibrosis, aggravating the pathological process of silicosis.

## 4 Discussion

Silicosis is a severe occupational hazard worldwide, particularly in China [19]. Once diagnosed, silicosis poses a tremendous psychological and social burden due to its incurability [20]. Nevertheless, the pathological characteristics and pathogenesis of silicosis remain unclear.

Many studies have shown a positive correlation between exposure to LPS and respiratory diseases including asthma-like symptoms, chronic airway obstruction, bronchitis, and increased respiratory responsiveness [21]. LPS exists in the air of Chinese coal mines, but it was also detected in the alveolar lavage fluid of silicosis patients, suggesting that LPS may play a critical role in the progression of silicosis. Therefore, the aim of the present study was to explore the relationship between exposure to LPS and silicosis.

Apoptosis refers to the orderly process of cell self-destruction in conjunction with inflammation under certain physiological or pathological conditions [22]. The apoptosis of AMs in silicosis has received much attention. Recent studies have demonstrated that LPS can alter the apoptotic activity via various signaling pathways. For example, LPS activation of the NF-κB-mediated signaling pathway promoted cell survival [23], whereas activation of the p38-mediated signaling pathway facilitated cell apoptosis [24]. However, there is limited support for increased apoptotic activity in AMs from silicosis patients following LPS stimulation.

Caspase-3 plays an essential role in mediating the intrinsic and extrinsic apoptotic pathways [25]. In the present study, the expression level of cleaved caspase-3 in AMs increased in both the silicosis patient and observer groups after LPS treatment. This finding suggests that all workers exposed to silica should pay attention to the presence of LPS in the surrounding environment. Since LPS exists in Asian sand dust [26], it may become attached to the free silica particles. Our study found that the cleaved caspase-3 level in the silica + LPS group was significantly higher than that in the silica group of mice with silicosis. These results suggest that LPS could exacerbate the apoptosis of AMs in silicosis.

Cytokines such as TNF-α, ILs, and transforming growth factor-β (TGF-β) are of great importance in the local pulmonary injury and inflammatory response of pulmonary fibrosis. IL-1β not only induces alveolar inflammation but also pulmonary interstitial fibrosis through the excessive repair of local injury [27,28]. IL-6 has the same inflammatory effects. Additionally, it has been reported that IL-6 promotes the expression of collagen, enhancing the TGF-β signaling pathway [29]. Moreover, TNF-α is involved in a wide range of inflammatory responses and silica-induced pulmonary fibrosis [30]. This study found that the concentrations of IL-1β, IL-6, and TNF-α were significantly higher in AMs treated with LPS than those in untreated AMs. These results imply that LPS could exacerbate the release of inflammatory factors in AMs in silicosis.

Previous research has observed an increased α-SMA mRNA level in the silicotic fibrosis model rat, indicating that α-SMA is closely related to the formation of pulmonary fibrosis [31]. Col-1 is a vital material basis for the entire fibrotic environment, and HYP is an important indicator of collagen tissue metabolism. Simultaneously, cellular nodules formed by the accumulation of silica-stimulated dust cells are the early form of silicon nodules. In the present study, we found that the levels of α-SMA, Col-1, and HYP were increased in mouse lung tissues following LPS treatment. In comparison with the silica group, there were infiltration and aggregation of several inflammatory cell types, the focal interstitial inflammation was further aggravated, and a greater number of cellular nodules appeared in the silica + LPS group. These results suggest that LPS could aggravate pulmonary fibrosis in silicosis. Normally, activated AMs will engulf invading silica dust; however, since they are unable to continuously dissolve silica dust, AMs become excessively activated and undergo apoptosis [32]. Excessively activated AMs release many inflammatory cytokines [33]. Subsequently, the released SiO_2_ is captured by other AMs, and this process is repeated, further inhibiting the repair process of lung tissue damage and eventually leading to irreversible fibrosis [34,35,36]. As the early pathological features of silicosis fibrosis, cellular nodules form gradually with the deepening degree of silica engulfment by AMs, inflammatory cell aggregation, and focal interstitial inflammation. They are composed of silica-phagocytosed macrophage aggregation and have no collagen fibers. Subsequently, fibroblasts appear and collagen fibers proliferate surrounding the nodules, forming cellular fibrous nodules or fibroblastic nodules in the lung tissue [37,38]. Thus, LPS or other substances that can increase apoptosis of AMs may be risk factors for silicosis.

In conclusion, LPS may accelerate the progression of silicotic pulmonary fibrosis by exacerbating the apoptosis of AMs. Therefore, greater emphasis should be placed on the effective protection against LPS in the silica-exposed environment in the future.

## References

[1] Bhattacharya S, Dey A, Pal A, Kar S, Saha S. Silicosis in the form of progressive massive fibrosis: a diagnostic challenge. Indian J Occup Environ Med. 2016;20:114–7. 10.4103/0019-5278.197548 PMC5299811 28194086

[2] Bang KM, Mazurek JM, Wood JM, White GE, Hendricks SA, Weston A. Silicosis mortality trends and new exposures to respirable crystalline silica-United States, 2001–2010. MMWR Morb Mortal Wkly Rep. 2015;64:117–20. PMC4584686 25674992

[3] Shafiei M, Ghasemian A, Eslami M, Nojoomi F, Rajabi-Vardanjani H. Risk factors and control strategies for silicotuberculosis as an occupational disease. N Microbes N Infect. 2018;27:75–7. 10.1016/j.nmni.2018.11.002 PMC6319297 30622714

[4] Bukovitz B, Meiman J, Anderson H, Brooks EG. Silicosis:diagnosis and medicolegal implications. J Forensic Sci. 2019;64:1389–98. 10.1111/1556-4029.14048 30901491

[5] Piera-Velazquez S, Mendoza FA, Jimenez SA. Endothelial to mesenchymal transition (EndoMT) in the pathogenesis of human fibrotic diseases. J Clin Med. 2016;5:45. 10.3390/jcm5040045 PMC4850468 27077889

[6] Arcangeli G, Cupelli V, Giuliano G. Effects of silica on human lung fibroblast in culture. Sci Total Environ. 2001;270:135–9. 10.1016/s0048-9697(00)00781-6 11327386

[7] Gozal E, Ortiz LA, Zou XY, Burow ME, Lasky JA, Friedman M. Silica-induced apoptosis in murine macrophages: involvement of tumor necrosis factor-alpha and nuclear factor-kappa B activation. Am J Respir Cell Mol Bio. 2002;27:91–8. 10.1165/ajrcmb.27.1.4790 12091251

[8] Joshi GN, Knecht DA. Silica phagocytosis causes apoptosis and necrosis by different temporal and molecular pathways in alveolar macrophages. Apoptosis. 2013;18:271–85. 10.1007/s10495-012-0798-y 23329178

[9] Chen S, Yuan JX, Yao SQ, Jin YL, Chen G, Tian W, et al. Lipopolysaccharides may aggravate apoptosis through accumulation of autophagosomes in alveolar macrophages of human silicosis. Autophagy. 2015;11:2346–57. 10.1080/15548627.2015.1109765 PMC4835201 26553601

[10] Mandrioli D, Schlunssen V, Adam B, Cohen RA, Colosio C, Chen W, et al. Who/ilo work-related burden of disease and injury: protocol for systematic reviews of occupational exposure to dusts and/or fibres and of the effect of occupational exposure to dusts and/or fibres on pneumoconiosis. Environ Int. 119;2018:174–85. 10.1016/j.envint.2018.06.005 29958118

[11] Feng FF, Cheng P, Zhang H, Li NN, Qi YX, Wang H, et al. The protective role of Tanshinone ILA model via TGF-β1/Smad signaling suppression, NOX4 inhibition and Nrf2/ARE signalin activation. Drug Des Dev Ther. 2019;13:4275–90. 10.2147/DDDT.S230572 PMC6930391 31908414

[12] Hou XM, Summer R, Chen ZY, Tian Y, Ma JJ, Cui J, et al. Lipid uptake by alveolar macrophages drives fibrotic responses to silica dust. Sci Rep. 2019;9:399. 10.1038/s41598-018-36875-2 PMC6344530 30674959

[13] Xu Q, Liu Y, Pan HH, Li Y, Yuan JL, Li P, et al. Aberrant expression of miR-125a-3p promotes fibroblast activation via Fyn/STAT3 pathway during silica-induced pulmonary fibrosis. Toxicology. 2019;414:57–67. 10.1016/j.tox.2019.01.007 30658076

[14] Ayaub EA, Kolb PS, Mohammed-Ali Z, Tat V, Murphy J, Bellaye P, et al. GRP78 and CHOP modulate macrophage apoptosis and the development of bleomycin induced pulmonary fibrosis. J Pathol. 2016;239:411–25. 10.1002/path.4738 27135434

[15] Son LY, Weng D, Dai WJ, Tang W, Chen S, Li C, et al. TH17 can regulate silica-induced lung inflammation through an IL-1β-dependent mechanism. J Cell Mol Med. 2014;18:1773–84. 10.1111/jcmm.12341 PMC4196653 25091058

[16] Reddy GK, Enwemeka CS. A simplified method for the analysis of hydroxyproline in biological tissues. Clin Biochem. 1996;29:225–9. 10.1016/0009-9120(96)00003-6 8740508

[17] Li WH, Wu HJ, Li YX, Pan HG, Meng T, Wang X. MicroRNA-143 promotes apoptosis of osteosarcoma cells by caspase-3 activation via targeting Bcl-2. Biomed Pharmacother. 2016;80:8–15. 10.1016/j.biopha.2016.03.001 27133034

[18] Srivastava AK, Khare P, Nagar HK, Raghuwanshi N, Srivastava R. Hydroxyproline: a potential biochemical marker and its role in the pathogenesis of different diseases. Curr Protein Pept Sci. 2016;17:596–602. 10.2174/1389203717666151201192247 26916157

[19] Zhao JQ, Li JG, Zhao CX. Prevalence of pneumoconiosis among young adults aged 24-44 years in a heavily industrialized province of China. J Occup Health. 2019;61:73–81. 10.1002/1348-9585.12029 PMC6499438 30698344

[20] Batista CRA, Gomes GF, Candelario-Jalil E, Fiebich BL, Oliveira AC. Lipopolysaccharide-induced neuroinflammation as a bridge to understand neurodegeneration. Int J Mol Sci. 2019;20:E2293. 10.3390/ijms20092293 PMC6539529 31075861

[21] Gioffre A, Marramao A, Gesu ID, Samele P, Paba E, Marcelloni AM, et al. Exposure to airborne endotoxin in Italian greenhouses: environmental analyses. Ind Health. 2018;56:150–4. 10.2486/indhealth.2017-0080 PMC5889933 29046490

[22] Huang XL, Feng Y, Xiong GQ, Whyte S, Duan J, Yang Y, et al. Caspase-11, a specific sensor for intracellular lipopolysaccharide recognition, mediates the non-canonical inflammatory pathway of pyroptosis. Cell Biosci. 2019;9:31. 10.1186/s13578-019-0292-0 PMC6438033 30962873

[23] Panday A, Inda ME, Bagam P, Sahoo MK, Osorio D, Batra S. Transcription factor NF-kappa B: an update on intervention strategies. Arch Immunol Ther Exp. 2016;64:463–83. 10.1007/s00005-016-0405-y 27236331

[24] Lv B, Huo F, Dang X, Xu ZG, Chen T, Zhang T, et al. Puerarin attenuates N-methyl-d-aspartic acid-induced apoptosis and retinal ganglion cell damage through the JNK/p38 MAPK pathway. J Glaucoma. 2016;25:e792–801. 10.1097/IJG.0000000000000505 27552519

[25] Communal C, Sumandea M, de Tombe P, Narula J, Solaro RJ, Hajjar RJ. Functional consequences of Caspase activation in cardiac myocytes. Proc Nati Acad Sci U S A. 2002;99:6252–6. 10.1073/pnas.092022999 PMC122935 11972044

[26] Ren YH, Ichinose T, He M, Song Y, Yoshida Y, Yoshida S, et al. Enhancement of OVA-induced murine lung eosinophilia by co-exposure to contamination levels of LPS in Asian sand dust and heated dust. Allergy Asthma Clin Immunol. 2014;10:30. 10.1186/1710-1492-10-30 PMC4058696 24982682

[27] Hutyrova B, Pantelidis P, Drabek J, Zurkova M, Kolek V, Lenhart K, et al. Interleukin-1 gene cluster polymorphisms in sarcoidosis and idiopathic pulmonary fibrosis. Am J Respir Crit Care Med. 2002;165:148–51. 10.1164/ajrccm.165.2.2106004 11790645

[28] Kobl M, Margetts PJ, Anthony DC, Pitossi F, Gauldie J. Transient expression of IL-1β induces acute lung injury and chronic repair leading to pulmonary fibrosis. J Clin Invest. 2001;107:1529–36. 10.1172/JCI12568 PMC200196 11413160

[29] Kusner LL, Young A, Tjoe S, Leahy P, Kaminski HJ. Perimysial fibroblasts of extraocular muscle, as unique as the muscle fibers. Invest Ophthalmol Vis Sci. 2010;51:192–200. 10.1167/iovs.08-2857 PMC2869047 19661226

[30] Tumelty KE, Smith BD, Nugent MA, Layne MD. Aortic carboxypeptidase-like protein (ACLP) enhances lung myofibroblast differentiation through transforming growth factor β receptor-dependent and -independent pathways. J Bio Chem. 2014;289:2526–36. 10.1074/jbc.M113.502617 PMC3908388 24344132

[31] Yu T, Li GH, Jia YM, Lou Y, Gan XY. Experimental study on the expression of α-SMA and HMGB1 in silicotic fibrosis model rats interfered by lumbricus. Zhonghua Lao Dong Wei Sheng Zhi Ye Bing Za Zhi. 2017;35:823–8. 10.3760/cma.j.issn.1001-9391.2017.11.005 29316752

[32] Lapp NL, Castranova V. How silicosis and coal workers’ pneumoconiosis develop – a cellular assessment. Occup Med. 1993;8:35–6. 8384379

[33] Yao SQ, He QC, Yuan JX, Chen J, Chen G, Lu Y, et al. Role of Fas/FasL pathway-mediated alveolar macrophages releasing inflammatory cytokines in human silicosis. Biomed Env Sci. 2013;26:930–3. 10.3967/bes2013.024 24331540

[34] Lim Y, Kim JH, Kim KA, Chang HS, Park YM, Ahn BY, et al. Silica-induced apoptosis in vitro and in vivo. Toxicol Lett. 1999;108:335–9. 10.1016/s0378-4274(99)00107-1 10511280

[35] Fan E, Fan J. Regulation of alveolar macrophage death in acute lung inflammation. Respir Res. 2018;19:50. 10.1186/s12931-018-0756-5 PMC5872399 29587748

[36] Thibodeau M, Giardina C, Hubbard AK. Silica-induced caspase activation in mouse alveolar macrophages is dependent upon mitochondrial integrity and aspartic proteolysis. Toxicol Sci. 2003;76:91–101. 10.1093/toxsci/kfg178 12857937

[37] Zhang Y, Li W, Zheng Y, Wang X, Li G, Yang H. Dynamic changes of pathological morphology and ultrastructure of lung injury in rats induced by SiO2 nanoparticles. Zhonghua Lao Dong Wei Sheng Zhi Ye Bing Za Zhi. 2014;32:504–10. 25182818

[38] Wang JS, Weng JF, Li X, Hu YB. Expression and role of sp1 in experimental silicotic fibrosis of rat. Ind Health Occup Dis. 2008;40:137–9.

